# Value of portal venous gas and a nomogram for predicting severe neonatal necrotizing enterocolitis

**DOI:** 10.1038/s41390-024-03605-6

**Published:** 2024-09-28

**Authors:** Yixian Chen, Yuhui Duan, Ba Wei, Yongjiang Jiang, Yadan Tan, Yijun Wei, Yuan Gan, Yujun Chen

**Affiliations:** 1 Department of Pediatrics, The Second Affiliated Hospital of Guangxi Medical University, Guangxi, China; 2 Neonatology, Liuzhou Hospital of Guangzhou Women and Children’s Medical Center, Guangxi, China; 3 Neonatology, The First People’s Hospital of Yulin, Guangxi, China; 4 Neonatology, Liuzhou Maternity and Child Healthcare Hospital, Guangxi, China

## Abstract

**Background:**

Whether portal venous gas (PVG) is a sign of severe neonatal necrotizing enterocolitis (NEC) and predicts poor prognosis remains uncertain.

**Methods:**

Patients from two centres were randomly assigned to a training set or a validation set. A nomogram model for predicting severe NEC was developed on the basis of the independent risk factors selected by least absolute shrinkage and selection operator (LASSO) regression analysis and multivariate logistic regression analysis. The model was evaluated based on the area under the curve (AUC), calibration curve, and decision curve analysis (DCA).

**Results:**

A total of 585 patients met the study criteria, and propensity score matching resulted in 141 matched pairs for further analysis. Patients with PVG had a greater risk of surgical intervention or death compared with patients without PVG. A prediction model for severe NEC was established based on PVG, invasive mechanical ventilation (IMV), serum platelet count (PLT) and pH <7.35 at the onset of NEC. The model had a moderate predictive value with an AUC > 0.8. The calibration curve and DCA suggested that the nomogram model had good performance for clinical application.

**Conclusion:**

A prediction nomogram model based on PVG and other risk factors can help physicians identify severe NEC early and develop reasonable treatment plans.

**Impact:**

- PVG is an important and common imaging manifestation of NEC.
- Controversy exists regarding whether PVG is an indication for surgical intervention and predicts poor prognosis.
- Our study suggested that patients with PVG had a greater risk of surgical intervention or death compared with patients without PVG.
- PVG, IMV, PLT and pH <7.35 at the onset of NEC are independent risk factors for severe NEC.
- A prediction nomogram model based on PVG and other risk factors may help physicians identify severe NEC early and develop reasonable treatment plans.

## Introduction

Necrotizing enterocolitis (NEC) is a common gastrointestinal infectious disease that affects new-borns, especially premature infants, with a morbidity rate ranging from 2 to 13% and a mortality rate of 29.3%.^1^ A total of 41.7% of children with NEC require surgical intervention, and NEC patients who require surgical intervention have a mortality rate as high as 34.9%.^2^ Survivors may have long-term complications, including growth impairment,^3^ neurodevelopmental delays, short bowel syndrome, intestinal strictures or adhesions, and cholestasis.^4^ Therefore, early detection, diagnosis, and treatment are crucial for improving the prognosis of NEC patients.

The aetiology and pathophysiology of NEC are multifactorial and currently not fully understood. Intestinal immaturity, formula feeding, intestinal dysbiosis, and infection are involved in the development of NEC.^5^ The diagnosis of NEC is based on nonspecific signs of gastrointestinal tract or systemic inflammation, local abdominal signs and imaging changes. Common imaging changes include intestinal dilation, intestinal stiffness, intestinal loop fixation, intestinal wall thickening, intramural gas, portal vein gas (PVG), ascites, and pneumoperitoneum.

In NEC, PVG is caused by the entrance of gas from gas-producing microorganisms within the intestinal cavity or an infected area into the intestinal wall vein and portal vein system, and PVG is considered an extension of intramural gas.^6^ PVG is not a fixed lesion of NEC and is usually a temporary change during disease progression that occurs over a short period.^7^ Pneumoperitoneum is an absolute indication for surgical intervention. However, in the absence of pneumoperitoneum, surgical intervention is necessary when medical treatment is ineffective and clinical manifestations, laboratory tests, or imaging findings indicate worsening of the condition.^8^ However, it is difficult to recognize surgical indications and identify the appropriate time for intervention when the situation continues to deteriorate. Previous studies have shown that PVG is a specific imaging change associated with widespread intestinal necrosis,^9^ which is a relative indication for surgical intervention and indicates poor prognosis.^10^ Other studies have shown that although PVG increases the surgical rate, it does not increase the NEC mortality rate, and no difference is noted in mortality between conservative and surgical treatment in children with PVG.^11^ Currently, there are few reports on the risk factors for NEC with PVG, and controversy exists regarding whether PVG predicts poor prognosis. Therefore, we conducted a retrospective and multi-centre analysis of the clinical data of hospitalized children with NEC to analyse the clinical characteristics of infants with NEC with PVG, established a scientific risk model for severe NEC and verified it. Least absolute shrinkage and selection operator (LASSO) regression is a popular method for variable selection. LASSO can produce a model with excellent performance and the fewest independent variables from the number of potential variables. Therefore, LASSO was utilized in our study to select variables related to severe NEC. This risk model aimed to predict surgery or death in infants with NEC, further improving the clinical identification of severe NEC, ensuring timely treatment, preventing further disease progression, and improving the long-term quality of life of these children.

## Methods

This retrospective analysis included 585 patients who were diagnosed with NEC at 2 centres in Guangxi, Liuzhou Maternity and Child Healthcare Hospital and The First People’s Hospital of Yulin (August 2013-August 2023). Patients were divided into a PVG group and a non-PVG group on the basis of whether a PVG was present during NEC. Patients who required surgical intervention or died were included in the severe NEC group, and all others were included in the moderate NEC group. Patients were included in this study if the following criteria were met: (1) the NEC onset time was during the neonatal phase, namely, at less than 28 days of gestation for term infants and at a corrected gestational age of less than 44 weeks for premature infants; (2) the diagnosis and treatment of NEC were performed according to modified Bell’s stage and Walsh grading criteria^12^ and the presence of stage ≥II NEC; and (3) patients with suspected NEC underwent a routine complete blood cell count, blood gas analysis, and abdominal imaging (plain film and/or ultrasound). The patients also underwent follow-up with plain abdominal film imaging and/or ultrasound after 4–6 h. Patients with congenital intestinal malformations, spontaneous intestinal perforation, meconium intestinal obstruction, or intestinal necrosis caused by other deformations were excluded from this study.

**Fig. 1:**
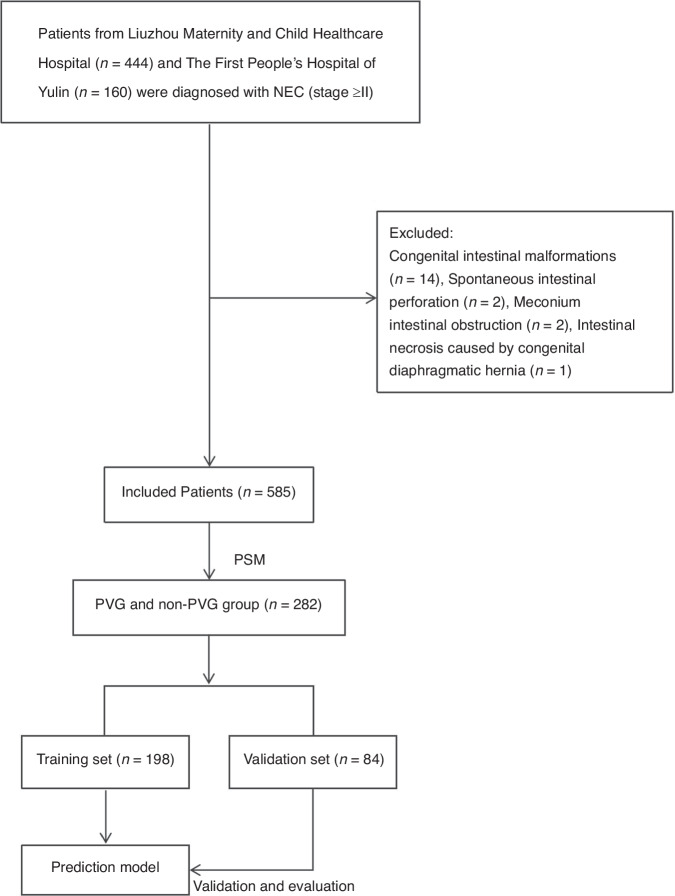
Flowchart of the study

Propensity score matching (PSM) analyses patients with PVG vs. non-PVG patients were performed via the 1:1 nearest neighbour method, with a calliper value of 0.02. Eighteen variables, including perinatal factors and demographic and baseline characteristics before NEC onset, were matched between the PVG group and the non-PVG group. Patients in the matched cohort were randomly divided into a training set and an internal validation set at a ratio of 7:3 to construct the predictive model and validation. This study was approved by the Liuzhou Maternity and Child Healthcare Hospital. A flowchart of the study design is shown in Fig. 1.

The clinical data were collected from electronic medical records during hospitalization. The data collected in this study included gestational age (GA), birth weight (BW), Apgar scores at 1 min and 5 min, sex, initiation of oxygen therapy, initiation of invasive mechanical ventilation (IMV), initiation of antibiotics, pulmonary surfactant (PS) therapy, blood transfusion, age at onset, and outcome of NEC. The perinatal factors included delivery method, conception method, gestational hypertension, chorioamnionitis, gestational diabetes mellitus (GDM), PROM, foetal distress, and antenatal corticosteroids. The laboratory parameters included the WBC count, PLT, pH, lactic acid (Lac) level, bacterial culture, and abdominal imaging results.

Variable definitions: (1) Foetal distress was diagnosed by an obstetrician before delivery and, in this study, was defined as the presence of abnormal decelerations not responsive to intrauterine resuscitative measures, including prolonged decelerations, repetitive moderate-to-severe variable decelerations or repetitive late decelerations.^13^ (2) PVG was defined as the accumulation of gas in the portal vein and its branches; PVG appeared as branching, linear, and radiative blood vessels that could extend from the main portal vein area to the periphery of both liver lobes on a supine abdominal plain film. On abdominal ultrasonography, echogenic lesions within the lumen can be observed moving with blood flow.^6^ (3) The indications for oxygen or IMV were based on the latest guidelines for neonatal oxygen therapy and MV during hospitalization. (4) An Apgar score ≤7 points at 1 min or 5 min was defined as asphyxia. (5) The enteral nutritional strategy for infants was based on the Chinese neonatal nutrition support guidelines.^14^ Human milk was the first choice for new-borns, followed by formula milk as the second choice. (6) Severe NEC was defined as the need for surgical intervention or the occurrence of death due to NEC.

SPSS (version 26) and R (version 4.4.1; R Foundation for Statistical Computing, Vienna, Austria) were used for data analysis. All the continuous variables were nonnormally distributed according to the Shapiro–Wilk test and are expressed as the median and interquartile range, and between-group differences were analysed via the Mann–Whitney U test. Categorical information is expressed numerically as percentages, and comparisons were performed via the chi-square test or Fisher’s exact test. *p* < 0.05 indicated statistical significance. First, univariate analysis that integrated various characteristics among groups was performed to identify potential factors responsible for severe NEC. Subsequently, LASSO regression analysis and multivariable logistic regression analysis were conducted to identify independent factors for predicting severe NEC. These variables were further applied to construct a nomogram. Finally, the new nomogram was verified with data from the validation set. With respect to the discriminative ability of the model, the receiver operating characteristic (ROC) curve and area under the curve (AUC) were calculated to evaluate its prediction accuracy. Calibration curves were generated to evaluate the predictive ability of the nomogram, and decision curve analysis (DCA) was performed to evaluate its clinical utility.

## Results

### PVG patient characteristics

Overall, PVG occurred in 153 patients (26.15%), 21.71% of whom underwent surgery, and 8.89% of whom died due to NEC. After PSM, a matched cohort with 141 pairs of patients with or without PVG emerged. No differences in perinatal factors, demographics, or baseline characteristics before the onset of NEC were noted between the two groups. Compared with patients without PVG, patients with PVG had higher rates of operation (41.8% vs. 16.3%) and death (16.3% vs. 7.8%). The patient characteristics are summarized in Table 1.

**Table 1:** Characteristics of non-PVG and PVG-NEC patients before and after matching.

	Before matching			After matching		
	Non-PVG (*n* = 432)	PVG (*n* = 153)	*P* value	Non-PVG (*n* = 141)	PVG (*n* = 141)	*P* value
Characteristics before NEC onset						
GA (weeks)	35.36 (31.29–38.29)	32.86 (29.79–37.07)	<0.001	33.29 (29.86–37.43)	33.43 (30–37.22)	0.996
BW (g)	2125 (1450–2895)	1660 (1255–2512.5)	0.001	1800 (1350–2455)	1770 (1300–2650)	0.931
Male	270 (62.5%)	88 (57.5%)	0.277	77 (54.6%)	84 (59.6%)	0.400
Asphyxia	66 (15.3%)	15 (9.8%)	0.092	10 (7.1%)	15 (10.6%)	0.295
Caesarean section	219 (50.7%)	73 (47.7%)	0.526	69 (48.9%)	67 (47.5%)	0.812
IVF-ET	39 (9.0%)	19 (12.4%)	0.228	21 (14.9%)	14 (9.9%)	0.206
Amniotic opacity	29 (6.7%)	6 (3.9%)	0.211	7 (5.0%)	6 (4.3%)	0.776
Foetal distress	68 (15.8%)	28 (18.3%)	0.469	21 (14.9%)	25 (17.7%)	0.519
Antenatal corticosteroids	114 (26.4%)	54 (35.3%)	0.036	45 (31.9%)	48 (34.0%)	0.704
GDM	81 (18.8%)	35 (22.9%)	0.271	33 (23.4%)	33 (23.4%)	1.000
Gestational hypertension	42 (9.7%)	19 (12.4%)	0.348	12 (8.5%)	17 (12.1%)	0.327
Chorioamnionitis	32 (7.4%)	10 (6.5%)	0.720	8 (5.7%)	9 (6.4%)	0.802
PROM ≥ 16 h	50 (11.6%)	19 (12.4%)	0.781	17 (12.1%)	18 (12.8%)	0.857
Initiation of oxygen therapy within 24 h after birth	150 (34.7%)	82 (53.6%)	<0.001	64 (45.4%)	70 (49.6%)	0.474
Initiation of IMV within 24 h after birth	58 (13.4%)	29 (19.0%)	0.099	20 (14.2%)	25 (17.7%)	0.416
Initiation of antibiotic within 1 week after birth	191 (44.2%)	93 (60.8%)	<0.001	78 (55.3%)	81 (57.4%)	0.719
PS	56 (13.0%)	27 (17.6%)	0.154	20 (14.2%)	24 (17.0%)	0.512
Blood transfusion	86 (19.9%)	28 (18.3%)	0.666	22 (15.6%)	25 (17.7%)	0.632
Characteristics at NEC onset						
Onset age (d)	9 (4–17)	11 (5–18.5)	0.157	8 (4–16.5)	11 (4.5–19)	0.184
WBC count (10^9^/L)	9.79 (6.76–13.25)	7.86 (4.96–12.17)	<0.001	9.92 (7.38–13.98)	7.86 (4.69–12.32)	0.001
PLT (10^9^/L)	281 (196.75–367.75)	254 (163–356.5)	0.100	277 (198.5–372)	251 (161.5–358.5)	0.199
pH <7.35	77 (17.8%)	57 (37.3%)	<0.001	28 (19.9%)	49 (34.8%)	0.005
Lac >2 mmol/L	135 (31.3%)	83 (54.2%)	<0.001	49 (34.8%)	74 (52.5%)	0.003
Septicaemia	17 (3.9%)	7 (4.6%)	0.732	4 (2.8%)	6(4.3%)	0.520
IMV	59 (13.7%)	55 (35.9%)	<0.001	20 (14.2%)	51 (36.2%)	<0.001
Operation	62 (14.4%)	65 (42.5%)	<0.001	23 (16.3%)	59 (41.8%)	<0.001
Death	28 (6.5%)	24 (15.7%)	0.001	11 (7.8%)	23 (16.3%)	0.028

*GA* gestational age, *BW* birth weight, *IVF-ET* in vitro fertilization-embryo transfer, *GDM* gestational diabetes mellitus, *PROM* premature rupture of membranes, *PS* pulmonary surfactant, *WBC* white blood cell, *PLT* platelet count, *pH* potential of hydrogen, *Lac* lactic acid, *IMV* invasive mechanical ventilation, *RBC* red blood cell.

### Baseline characteristics

The training set consisted of 198 neonates, including 130 neonates with moderate NEC and 68 neonates with severe NEC. In the training set, univariate analysis revealed that GA, PS, WBC, PLT, pH <7.35, Lac >2, IMV and PVG were significantly associated with severe NEC (*p* < 0.05) (Table 2).

**Table 2:** Univariate analysis of severe NEC.

.	Moderate NEC (*n* = 130)	Severe NEC (*n* = 68)	*P* value
GA (weeks)	33.86 (30.40–37.57)	32 (29.43–35.5)	0.033
BW (g)	1825 (1350–2612.5)	1675 (1245–2400)	0.327
Male	72 (55.4%)	44 (64.7%)	0.206
Asphyxia	11 (8.5%)	10 (14.7%)	0.175
Caesarean section	69 (53.1%)	30 (44.1%)	0.231
IVF-ET	18 (13.8%)	6 (8.8%)	0.304
Foetal distress	24 (18.5%)	11 (16.2%)	0.689
Antenatal corticosteroids	48 (36.9%)	20 (29.4%)	0.291
GDM	32 (24.6%)	18 (26.5%)	0.775
Gestational hypertension	18 (13.8%)	4 (5.9%)	0.090
Chorioamnionitis	9 (6.9%)	3 (4.4%)	0.697
PROM ≥ 16 h	18 (13.8%)	9 (13.2%)	0.905
Initiation of oxygen therapy within 24 h after birth	59 (45.4%)	34 (50.0%)	0.537
Initiation of IMV within 24 h after birth	17 (13.1%)	15 (22.1%)	0.103
Initiation of antibiotic within 1 week after birth	72 (55.4%)	39 (57.4%)	0.791
PS	16 (12.3%)	16 (23.5%)	0.042
Blood transfusion	21 (16.2%)	16 (23.5%)	0.206
Onset age (d)	9 (4–17)	10 (5–17.75)	0.431
WBC count (10^9^/L)	9.69 (7.04–13.88)	7.03 (4.14–11.85)	0.003
PLT (10^9^/L)	311.5 (203.75–395.75)	205.5 (115–319.75)	<0.001
pH <7.35	17 (13.1%)	35 (51.5%)	<0.001
Lac >2 mmol/L	44 (33.8%)	40 (58.8%)	0.001
Septicaemia	4 (3.1%)	5 (7.4%)	0.311
IMV	10 (7.7%)	36 (52.9%)	<0.001
PVG	53 (40.8%)	50 (73.5%)	< 0.001

### Variable selection and predictive model establishment

**Fig. 2:**
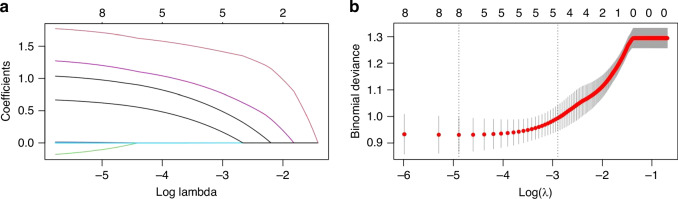
Variable selection using the LASSO binary logistic regression model. The optimal lambda was used to select five variables with nonzero coefficients. Each line represents a parameter, and the end of the parameter corresponds to a vertical coordinate, which is the coefficient (**a**). LASSO coefficient profiles for the five features were plotted against log (lambda) sequences. Two vertical dotted lines were plotted on the basis of the positive and negative standard error thresholds of log(lambda) (**b**).

**Fig. 3:**
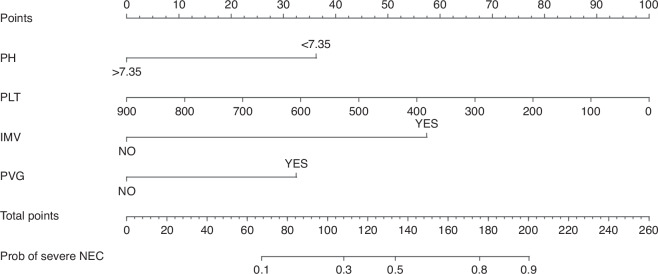
Nomogram for predicting severe NEC. Each score of the variable was summed to obtain the total score on the total points axis, which corresponds to the probability of severe NEC.

Eight variables from the univariate analysis of the training set were analysed using the LASSO model. A coefficient profile plot and a cross-validated error plot of the LASSO regression results are shown in Fig. 2. Five variables were identified on the basis of the 1 standard error of the minimum value chosen as the optimal λ and used for multivariate logistic regression. Four variables were identified as independent risk factors at the onset of NEC: PVG (OR: 2.922, 95% CI = 1.371–6.225), IMV (OR: 6.666, 95% CI = 2.767–16.057), PLT (OR: 0.996, 95% CI = 0.994–0.999) and pH <7.35 (OR: 3.309, 95% CI = 1.462–7.489). A novel nomogram incorporating these four variables was built to predict severe NEC (Fig. 3).

### Validation and evaluation of the prediction model

**Fig. 4:**
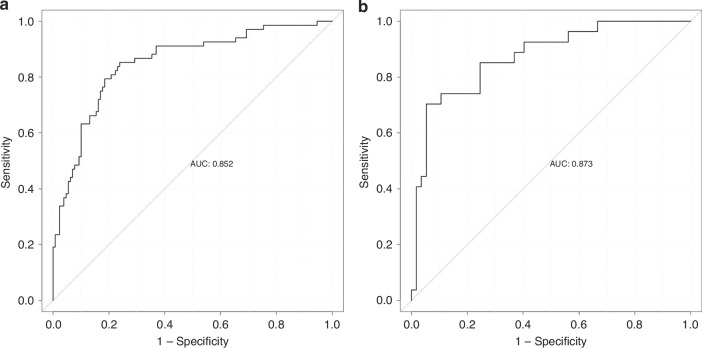
ROC curves. The discriminative ability of the nomogram was measured and compared according to AUC for the training set (**a**) and validation set (**b**).

The ROC curves of the nomogram are compared in Fig. 4. The AUCs of the training set and the validation set were 0.852 (95% CI = 0.794–0.910) and 0.873 (95% CI = 0.792–0.955), indicating strong model accuracy (Fig. 4).

**Fig. 5:**
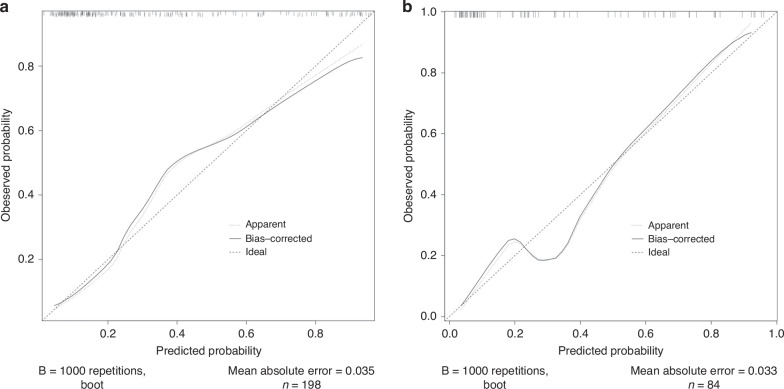
Calibration curves. The closer the curves were to the standard diagonal dotted line, the more consistent the predicted probabilities were with the actual probabilities. The diagonal dotted line represents a perfect prediction by an ideal model. The consistency of the predicted and actual probabilities of the training set (**a**) and validation set (**b**).

The calibration curves (Fig. 5) revealed good agreement between the predicted and actual severe NEC probabilities. The Hosmer‒Lemeshow test demonstrated excellent agreement between the calculated and observed probabilities in the training set (*p* = 0.541) and validation set (*p* = 0.759).

**Fig. 6:**
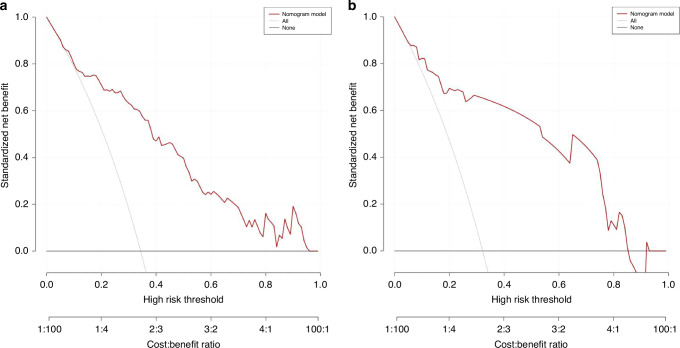
DCA curves. The y-axis represents the net benefit. The x-axis represents the potential threshold probability for severe NEC. The thick solid line represents all patients without severe NEC, the thin solid line represents all patients who developed severe NEC, and the red line represents the nomogram. The net benefit of the nomogram in the training set (**a**) and validation set (**b**).

The DCA curve (Fig. 6) confirmed the favourable clinical validity of the model. In summary, these results validate the accuracy and reliability of the nomogram and indicate that the nomogram might be useful in clinical practice.

## Discussion

NEC is a common infectious disease that affects preterm infants during the neonatal period. With improvements in the technology used for the care of premature infants, the incidence and mortality rate of NEC have decreased;^15^ however, some studies have shown that the incidence of NEC increases proportionally with the survival rate of premature infants.^16^ The surgical and mortality rates of NEC are high, and survivors may experience long-term complications. The relevant data reported by different countries and regions may vary. In our study, 21.71% of stage ≥II NEC patients required surgical intervention, and the mortality rate was 8.89%.

PVG is an important imaging manifestation of NEC and occurs in approximately 10%-30% of NEC patients. In Dordelmann et al.’s study, abdominal ultrasound PVG showed 86% specificity and 45% sensitivity in diagnosing NEC stage ≥II.^17^ In our study, PVG was observed in 26.15% (153/585) of patients with stage ≥II NEC. Differences in the characteristics between the NEC patients with and without PVG before and at NEC onset were noted. In the PVG group, NEC patients had a lower GA and BW than non-PVG patients did, and the GAs were 32.86 (29.79–37.07) weeks and 35.36 (31.29–38.29) weeks, respectively. This finding differed from those of previous studies. In Sharma et al.’s study, PVG was observed in 33% (64/194) of patients with stage ≥II NEC and was less likely to occur among infants with lower GAs than among infants without PVG (30.8 ± 4 vs. 29.3 ± 4.2 weeks).^11^ However, no differences in BW (1609 ± 761 vs. 1434 ± 810 g) were noted. PVG can be caused by umbilical vein catheterization, cow milk protein allergy, hypertrophic pyloric stenosis, convulsions, or respiratory system diseases, and it is necessary to differentiate NEC from these diseases when there are no typical clinical manifestations of NEC in clinical practice.^18^ PVG is considered an extension of intramural gas in NEC and is a specific sign of panintestinal necrosis, with 34% sensitivity and 94% specificity according to Tam et al.^6,9^ However, in one case report, PVG indicated early-stage term NEC without typical clinical manifestations.^7^ Controversy exists regarding whether PVG indicates a poor prognosis. According to previous studies, PVG is an independent risk factor for the rapid progression of NEC^18^ and is associated with a greater probability of acute peritonitis and subsequent shock,^11^ surgical intervention,^17^ and death.^9^ However, a meta-analysis revealed no significant correlation between PVG and surgical intervention or death.^19^ In our study, PSM was utilized to control for the confounding factors of PVG and severe NEC. Compared with NEC patients without PVG, NEC patients with PVG had higher rates of surgery and mortality before and after matching (41.8% vs. 16.3% and 16.3% vs. 7.8%, respectively, in the matched cohort). Similar results were shown in Sharma et al.’s study, with higher rates of surgery observed in NEC patients with PVG than in those without PVG (48% vs. 27%);^11^ however, there was no difference in NEC mortality. PVG increased the risk of subependymal haemorrhage and intestinal necrosis but does not increase the occurrence of intestinal stricture, obstruction, or short bowel syndrome.^20^ PVG was an independent risk factor for severe NEC (surgery or death) in our study, and the other independent risk factors included IMV, PLT and pH<7.35 at NEC onset. A prediction model for severe NEC based on the above independent risk factors was established, and a risk nomogram for severe NEC was generated.

NEC results from a combination of multiple factors. Premature infants are more susceptible to harmful factors because of their immature gut. Hypoxia is an important initiating factor for intestinal damage, and other factors, such as infection, formula milk, and the gut microbiota, can work together to cause NEC.^21^ The pathological changes in NEC are not limited to the gut, as bacteria and their toxins can enter the systemic circulation through the damaged intestinal barrier, resulting in a systemic inflammatory response and even multisystem organ failure.^22^ Dolgin et al. reported that respiratory changes preceded direct gastrointestinal signs of NEC.^23^ In the early stages of NEC, patients often exhibit unexplained signs of respiratory compensation and decompensation and require increased respiratory support. Our study suggested that IMV at the onset of NEC was more likely to occur in patients with PVG and increased the risk of severe NEC (OR: 6.666). IMV during NEC episodes is usually caused by frequent apnoea and respiratory failure, and IMV helps improve intestinal hypoxia.

Regarding laboratory indicators, haematological abnormalities occur prior to the time at which NEC is diagnosed, and these abnormalities are valuable for diagnosing and evaluating the severity and prognosis of NEC. According to a study by Shi et al., a WBC count >20 ×10^9^/L or <5 ×10^9^/L and a CRP > 50 mg/L are independent risk factors for surgical NEC.^24^ Previous studies have confirmed the value of the PLT in predicting surgical intervention and mortality in patients with NEC.^25,26^ Ververidis et al. reported that the lowest PLT during NEC was lower in patients with stage III NEC than in patients with stage II NEC, and similar results were observed between patients who died and those who survived. Severe thrombocytopenia (<100 ×10^9^/L) has a sensitivity of 69%, specificity of 60%, and positive predictive value of 89% in predicting intestinal gangrene.^27^ Therefore, closely monitoring the trend of PLT changes during NEC is helpful for the early identification of severe NEC. In our study, we did not find a significant difference in the PLT between the PVG and non-PVG groups, which is consistent with the results of Lin et al.^20^ Significant differences in CRP, fibrinogen degradation products (FDP and D-dimer), and blood glucose were reported in the study by Lin et al.^20^; however, our study did not include these variables. We found a significant difference in PLT between the severe and moderate NEC groups, at 311.5 (203.75–395.75) and 205.5 (115–319.75), respectively. The impact of severe thrombocytopenia with a score of 90 to 100 according to the nomogram was greater than that of the other three factors according to the prediction model of severe NEC. Platelet consumption may be due to platelet activation by bacterial products, leading to microvascular aggregation and the formation of microthrombi in the affected intestine.^28,29^ The role of acidosis in the prognosis of NEC patients has long been clarified. Lin et al. reported that, compared with patients with NEC without PVG, patients with PVG had lower pH values and higher lac values at NEC onset.^20^ In this study, patients in the PVG group had higher rates of pH <7.35 and Lac >2 at the onset of NEC, which is consistent with the findings of Lin et al. The pH decreased 3 days before onset in a previous study.^30^ Miner et al. confirmed that the pH was lower in stage III NEC than in stage II NEC before disease onset and that this indicator could also predict death.^31^ Other studies have shown that low pH during the acute phase of NEC, especially persistent acidosis (pH <7.2), is an independent risk factor for surgical intervention.^32,33^ Our study reached a similar conclusion, namely, that a pH <7.35 was an independent risk factor for severe NEC.

A systematic review revealed the protective effect of caesarean section on the development of NEC.^34^ Riskin et al. reported that caesarean section was a risk factor for NEC in multiple pregnancies; however, no significant correlation with NEC was noted in singleton pregnancies.^35^ Our study revealed that caesarean section did not increase the risk of NEC. The same review also indicated that PS therapy was a controversial risk factor for NEC. In our study, PS usage was greater in the severe NEC group than in the moderate NEC group, at 23.5% and 12.3%, respectively. However, multiple factor analysis revealed that PS treatment did not increase the risk of severe NEC. Other risk factors that were related to severe NEC in previous studies, such as shock, volumetric onset,^36^ hypothermia,^23^ haemodynamically significant patent ductus arteriosus,^37^ and coagulation disorders,^38^ were not included in this study, and this was due to the limited sample size utilized.

## Conclusion

The presence of PVG in infants with NEC may indicate aggravation of the condition, which increases the risk of surgical intervention and death. We developed a prediction model for severe NEC using PVG, IMV, PLT and pH<7.35 at the onset of NEC as risk factors. Using a nomogram to intuitively express risks may be helpful for the early identification of severe NEC and the development of reasonable treatment plans.

## Data Availability

All the data generated or analysed during this study are included in this published article.
